# The role of indocyanine green fluoroscopy for intraoperative bile duct visualization during laparoscopic cholecystectomy: an observational cohort study in 70 patients

**DOI:** 10.1186/s13037-019-0182-8

**Published:** 2019-01-12

**Authors:** Peter C. Ambe, Jens Plambeck, Victoria Fernandez-Jesberg, Konstantinos Zarras

**Affiliations:** 1 Department of Visceral, Minimally Invasive and Oncologic Surgery, Marien Hospital Düsseldorf, Rochusstr. 2, 40479, Düsseldorf, Germany; 2 Department of Medicine Faculty of health, Witten / Herdecke University, Witten, Germany

**Keywords:** Indocyanine green, Laparoscopic cholecystectomy, Bile duct injury, Intraoperative fluorescence angiography

## Abstract

**Background:**

Bile duct injury is the most feared complication during laparoscopic cholecystectomy. Real-time intraoperative imaging using indocyanine green (ICG) might reduce the risk of bile duct injury by improving visualization of the biliary tree during laparoscopic cholecystectomy. We compared the outcomes of laparoscopic cholecystectomy in patients with and without real-time ICG.

**Methods:**

A retrospective analysis of the data of patients undergoing laparoscopic cholecystectomy with and without ICG in a referral centre for minimally invasive surgery was performed. We hypothesized that laparoscopic cholecystectomy with real-time ICG enables a better identification of the biliary tree and thus increases surgical safety. The outcomes of laparoscopic cholecystectomy with and without ICG were compared using the duration of surgery, the rate of bile duct injury, the rate of conversion, complications and the length of stay.

**Results:**

Seventy patients including 29 with and 41 without ICG underwent laparoscopic cholecystectomy within the period of investigation. The median duration of surgery was 53.0 vs. 54.0 min while the median length of stay was 2.0 d in the group with and without ICG respectively. The rate of conversion was 2.4% in the group without ICG, while no conversion was performed in the group with ICG. NO bile duct injury occurred in both groups. These differences were not statistically significant.

**Conclusion:**

Laparoscopic cholecystectomy with real-time indocyanine green fluorescence cholangiography enables a better visualization and identification of biliary tree and therefore should be considered as a means of increasing the safety of laparoscopic cholecystectomy.

## Introduction

Cholecystectomy is one of the most commonly performed procedures in general surgery with over a million procedures worldwide each year [1]. The minimal invasive access, notably laparoscopic route now represents the standard procedure for patients with benign gallbladder disorders. Bile duct injury with varying severity represents the most feared complication following laparoscopic cholecystectomy [2]. The lifetime risk bile duct injury following laparoscopic cholecystectomy for gallbladder stones without acute inflammation in the hands of an experienced surgeon has been reported to be about 0.4% [3]. Laparoscopic cholecystectomy for acute cholecystitis has been shown to be associated with a much higher risk of bile duct injury with rates as high as 4% being reported in the literature depending on the extent of gallbladder inflammation [4, 5]. Although a large portion of bile duct injury consist of minor injuries, extensive and complex injuries to the biliary tree during laparoscopic cholecystectomy might have devastating consequences for the patients involved [6, 7].

Many measures have been implemented to reduce the risk of bile duct injury during laparoscopic cholecystectomy. The critical view of safety and dissection within the triangle of calot constitute the most commonly employed means of prevention or reducing bile duct injury [8]. Intraoperative sonographic and radiographic examination of the biliary tree constitute standard methods for studying the biliary anatomy during surgery [9, 10]. Despite these measures, bile duct injury still remains a serious problem.

Intraoperative studies of the biliary tree have been achieved using Indocyanine Green ICG [11]. ICG is a water soluble tricarbocyanine molecule that is almost completely protein bound following intravenous injection. ICG is metabolized by the liver and excreted in bile [12, 13]. The angiographic feature of ICG is based on its fluorescent character in the near-infrared range between 790 and 805 nm, which can be detected by specialized infrared video cameras. The fact that ICG is metabolized in the liver and excreted via bile makes it an excellent medium for biliary tree imaging [14–16].

Herein we report our experience with lifetime IGC imaging of the biliary tree in patients undergoing laparoscopic cholecystectomy for benign gallbladder disorders and compared the outcomes of patients undergoing laparoscopic cholecystectomy with and without ICG.

## Methods

This is an analysis of prospectively data of patients undergoing laparoscopic cholecystectomy for benign gallbladder diseases in a referral centre for visceral, minimally invasive and oncologic surgery. The indication for surgery was either symptomatic gallbladder stones or acute cholecystitis. The preoperative diagnostic was performed as reported elsewhere [17]. All procedures were performed as in-hospital procedures in general anesthesia. Ethic approval for this study was waived following consultation with the institutional review board because all patients consented on the use of their data in this study.

Laparoscopic cholecystectomy in our centre is performed using the three port technique. Surgery begins with an infra-umbilical incision and peritoneum is instilled via a veress needle. The intraabdominal pressure is initially set at 20 mmHg. Two 5 mm ports are introduced in the right upper quadrant under visual control. Hereafter, the intraabdominal pressure is reduced to 14 mmHg. The triangle of calot is bluntly dissected to reveal the cystic duct and the cystic artery which are divided between clips. This is followed by the dissection of the gallbladder off the liver. The gallbladder is removed from the abdomen using retrieval bag.

**Fig. 1:**
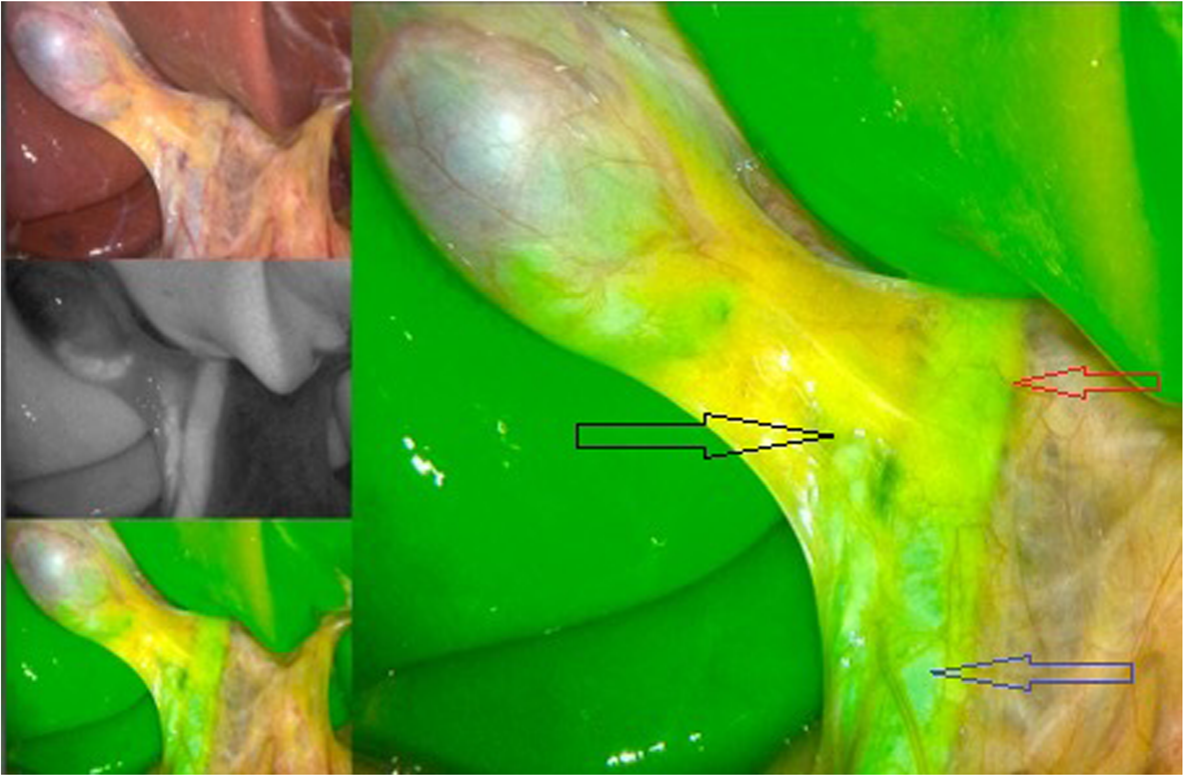
Intraoperative imaging after application of 0.5 ml of ICG. Note the cystic duct and the main bile duct. Red arrow: ductus hepaticus communis, black arrow: ductus cysticus, blue arrow: ductus choledochus

**Fig. 2:**
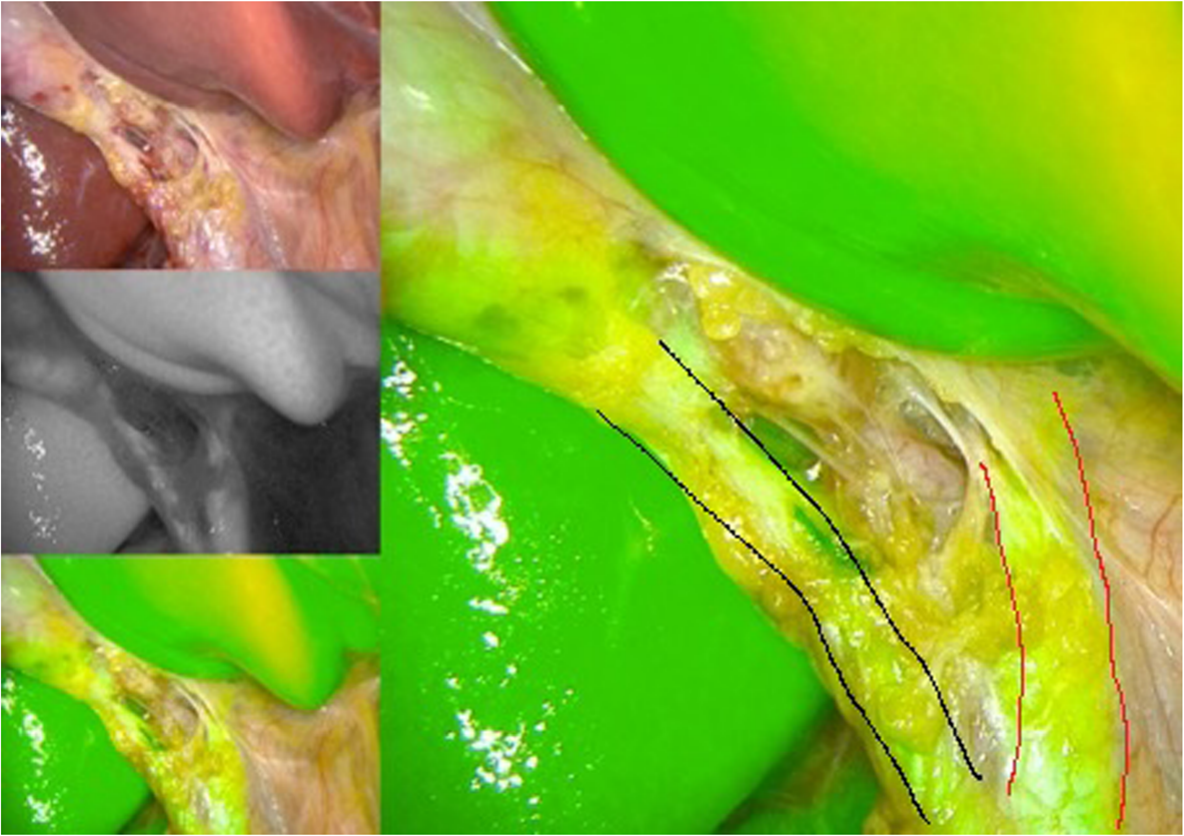
Intraoperative imaging with Indocyanine green. Note the clear – cut differentiation between cystic duct and cystic artery. Red lines: ductus hepaticus communis, black lines: ductus cysticus

**Fig. 3:**
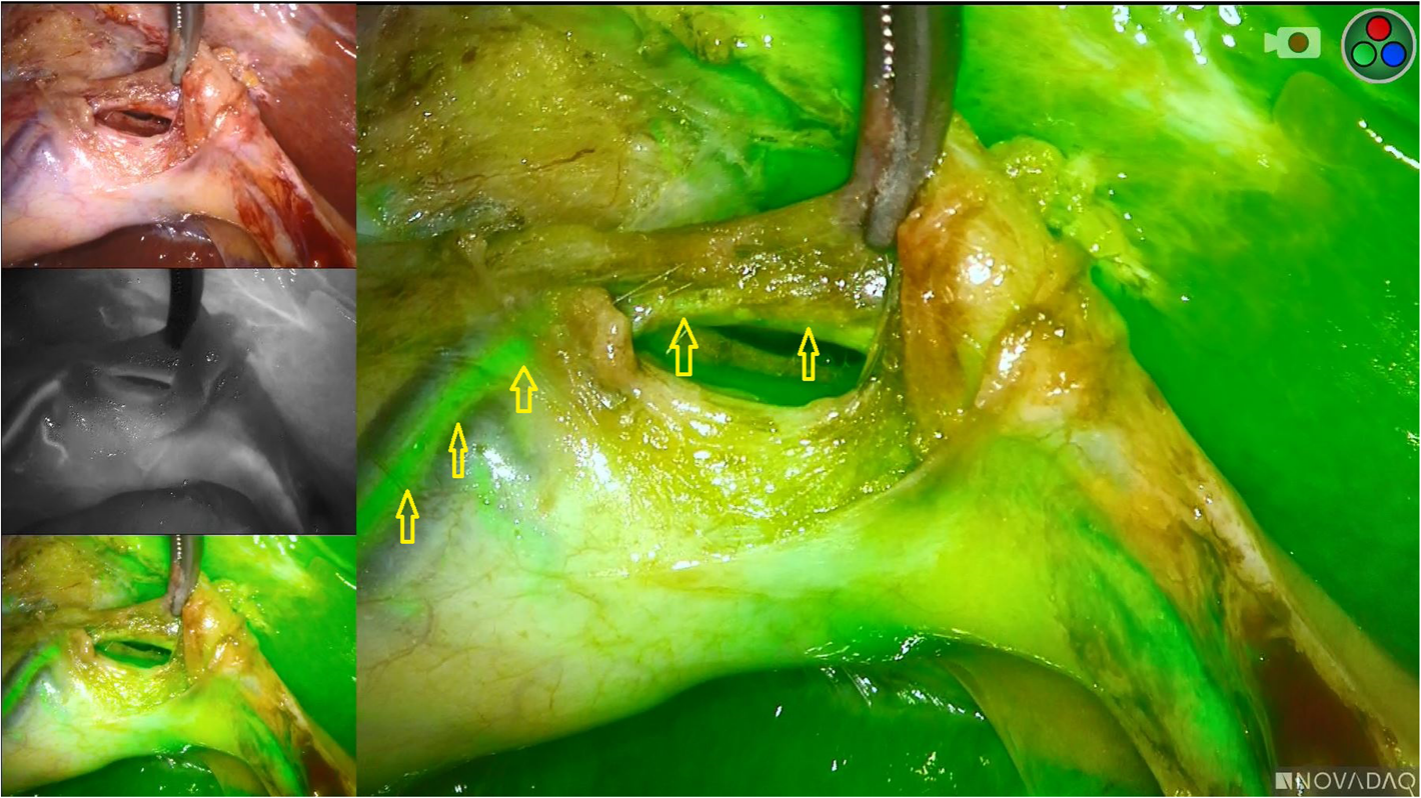
Indocyanine green fluorescence angiography showing a fluorescent cystic artery. Yellow arrows: cystic artery

**Fig. 4:**
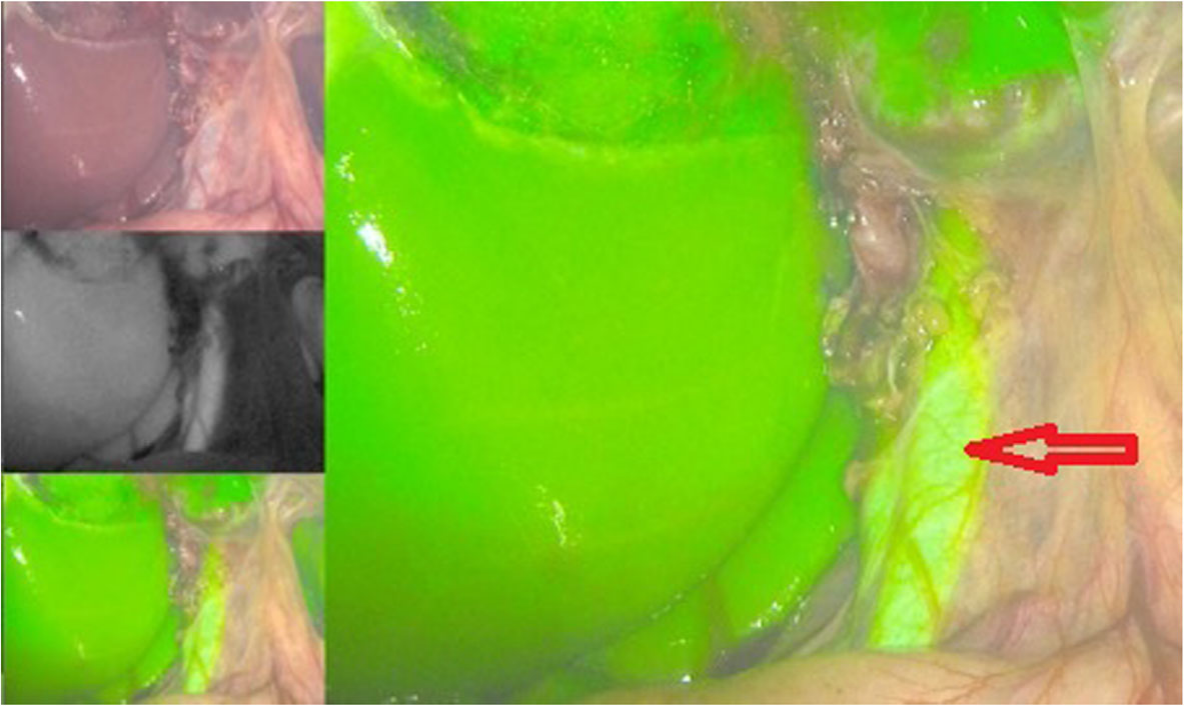
Documentation of the main bile duct at the end of gallbladder dissection. Red arrow: ductus hepaticus communis

Prior to February 2017, laparoscopic cholecystectomy was performed without routine intraoperative imaging of the biliary tree. Beginning February 2017 laparoscopic cholecystectomy was performed with ICG. 0.5 ml of ICG was given via intravenous infusion one hour prior to surgery. Fluorescence imaging was performed using the PINPOINT endoscopic fluorescence imaging system (Novadaq, Canada). The triangle of calot was exposed to display the biliary tree, Fig. 1. This is followed by dissection of the triangle of calots to expose the cystic duct and artery, Fig. 2. Additional, the cystic artery can be independently visualized a few minutes following ICG injection, Fig. 3**.** Hereafter, both structures are safely clipped and divided. The gallbladder is then dissected off the liver as usually, Fig. 4.

**Fig. 5:**
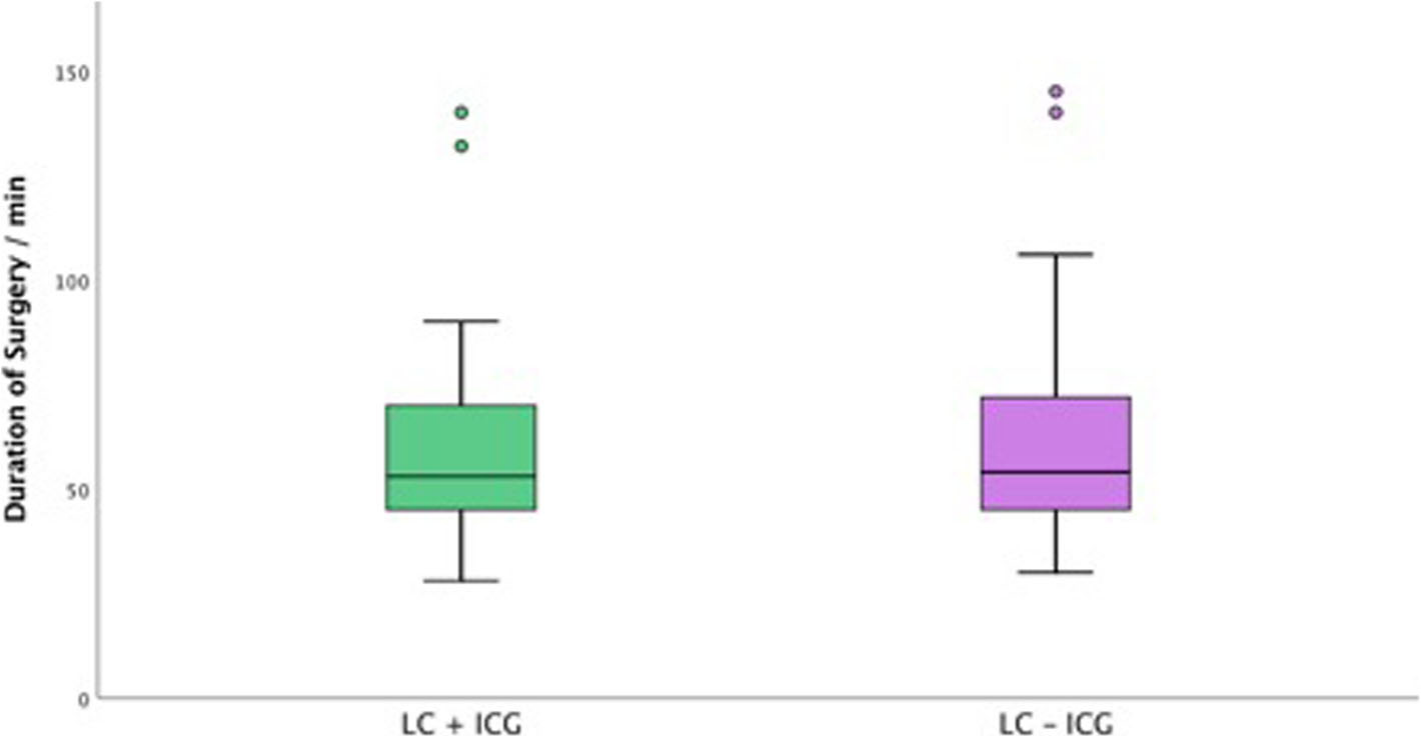
Duration of Surgery. There was no statistically significant difference amongst patients under laparoscopic cholecystectomy with and without Indocyanine green

The charts of all consecutive patients managed by a single surgeon (KZ) within the period of investigation were retrospectively reviewed. Baseline data including sex, age and body mass index (BMI) were extracted in all cases. Perioperative data including indication for surgery, relevant medical conditions characterized using the American Society of Anesthesiologists score (ASA), the duration of surgery, conversion from laparoscopic to open cholecystectomy, postoperative complications and length of stay were noted.

Prospectively collected data of patients undergoing laparoscopic cholecystectomy with ICG from February 2017 till December 2017 were compared to those of a retrospective group undergoing laparoscopic cholecystectomy without ICG managed between January 2016 and January 2017. Only patients undergoing laparoscopic cholecystectomy were included for analysis. Patients undergoing open surgery or simultaneous cholecystectomy during surgery for other reasons were excluded.

Primary outcomes included the duration of surgery defined as the time from first incision to suture, conversion following attempted laparoscopic cholecystectomy and bile duct injury. The length of stay and postoperative complications constituted our secondary outcome measures. The length of stay was defined as the time interval between surgery and discharge in days.

To eliminate the effect of surgical expertise on the defined outcomes, only procedures performed by a single surgeon (KZ) were analyzed.

The data gathered was analyzed using the Statistical Package for Social Science (SPSS, IBM version 25). Since the data was not normally distributed, continuous variables were described using absolute case numbers and percentages while central tendencies were reported using medians with the corresponding ranges. Analytic statistics was performed using the chi square test or the Mann – Withney - U test were necessary. All calculations were done with a 95% confidence interval. The two-sided *p*-value was reported in all cases and statistical significance was set at *p* < 0.05.

## Results

Seventy patients undergoing laparoscopic cholecystectomy for benign gallbladder disorders managed by a single surgeon within the period of investigation from December 2015 to December 2017 were included for analysis. The study group included 29 cases of laparoscopic cholecystectomy with ICG while the control group consisted of 41 consecutive cases of laparoscopic cholecystectomy without ICG performed by the same surgeon prior to introduction of ICG. Both groups were comparable with regard to demographic and perioperative characteristics, Table 1.

**Table 1:** Summary of the demographic and perioperative features of the study population

Characteristics	LC without ICG	LC with ICG	*P*-value
Sex			
Female	16 (39.0%)	14 (48.3%)	0.44
Male	25 (61.0%)	15 (51.7%)	
Age			
Median	56.0 yrs	61.0 yrs	0.19
Range	29–85 yrs	12–91 yrs	
ASA Score			
1–2	23 (56.1%)	17 (58.6%)	
> 2	18 (43.9%)	12 (41.4%)	0.78
Duration of Surgery			
Median	54.0 min	53.0 min	0.40
Range	30–145 min	28–140 min	
Lenght of Stay			
Median	2.0 d	2.0 d	0.57
Range	2–13 d	2–17 d	

*d* days, *min* minutes, *yrs* years, *ASA* American Society of Anesthesiologists, *LC* Laparoscopic cholecystectomy, *ICG* Indocyanine green

Symptomatic cholecystolithiasis was the indication for surgery in 82.9% (58 cases), while surgery was performed due to acute cholecystitis in 17.1% (12 cases). Acute cholecystitis was managed in 13.8% of cases in the group with ICG compared to 19.5% of cases in the group without ICG. This difference was not statistically significant, *p* = 0.53.

The duration of surgery in both groups is presented in Fig. 4. There was no statistically significant difference amongst both groups with regard to the duration of surgery, Fig 5. Conversion from laparoscopic to open cholecystectomy was performed in one case (2.4%) in the group without ICG, while no conversion was performed in the group with ICG. The median length of stay was two days in both groups. No relevant complications including bile duct injury were documented in both groups.

## Discussion

Laparoscopic cholecystectomy with and without intraoperative fluorescence studies with ICG was investigated in this study. No significant differences were recorded amongst patients undergoing laparoscopic cholecystectomy with or without ICG with regard to all outcome measures including the duration of surgery, rate of bile duct injury, the rate of conversion and length of stay. No injection-related and surgical complications were recorded.

The duration of surgery and the rate of conversion from laparoscopic to open cholecystectomy represent outcome measures that have been frequently used to indirectly access the surgical challenge during laparoscopic cholecystectomy. We postulated that the use of ICG during laparoscopic cholecystectomy enables a better, easier and faster identification of the biliary tree anatomy thereby increasing the safety of cholecystectomy by reducing the risk of bile duct injury. Besides, early and easy identification of biliary anatomy could facilitate the dissection in the triangle of calot thereby reducing the duration of surgery.

Bile duct injury is the most feared complication following laparoscopic cholecystectomy. This complication has been shown to occur even in the hands of surgeons with profound expertise in LC. In fact the lifetime risk of bile duct injury has been estimated at 0.4% [3]. As such there is always some substantial risk of bile duct injury whenever laparoscopic cholecystectomy is performed. Consequently, the need to maximize surgical safety and reduce the risk of complications cannot be overemphasized.

ICG is being increasingly used during laparoscopic cholecystectomy to better understand the anatomy of the biliary tree and prevent or reduce the risk of bile duct injury. This technique has been proven both in animal models and in clinical setting to be safe and effective [9, 18, 19]. Unlike in this series, most of the available studies on laparoscopic cholecystectomy with ICG so far did not contain a control group of patients undergoing standard LC without ICG [13, 20, 21]. The results of the ongoing multicenter FALCON trail in the Niederlands designed to investigate the outcomes of laparoscopic cholecystectomy with and without ICG might provide more insight in future [22].

In a recently published study on robotic cholecystectomy by Buchs et al., ICG contributed to a significant reduction in the duration of surgery compared to cases without ICG [23]. This trend could not the confirmed in our series. Recently, Gangemi et al. [24] reported that the use of ICG during laparoscopic cholecystectomy was associated with a significant reduction in the rate of conversion from laparoscopic to open cholecystectomy. Only one conversion was recorded in our study making an interpretation unreasonable.

The findings from our study with regard to the duration of surgery and the rate of conversion are not surprising considering the extensive expertise in laparoscopic surgery in our centre of excellence in minimally invasive surgery. More so, all cases were managed by the most experience member of the surgical team (KZ). It is therefore questionable, if similar results would be generated if procedures performed by other members of the surgical team including residents were to be analyzed.

Although all cases included in this series were consecutively recruited, selection bias must be discussed as a major limitation to this study. Besides, only patients managed by a single experienced surgeon were included for analysis. This constitutes a serious bias as it is not clear whether or not similar results would be generated by a less experienced surgeon. As stated above, it is unclear, if similar results would be generated in an unselected population. More so, the results might be subjected to our departmental standards including but not limited to the three port technique. These results therefore cannot be readily projected on other institutions. The relative small size of the study population must be stated as a limitation to this study. The need for larger and well-designed studies on this topic cannot be overemphasized.

Taken together, the results from this series did not show any difference in outcomes between patients undergoing laparoscopic cholecystectomy with and without ICG. While this trend must be argued with the availability of profound expertise in minimally invasive surgery at our institution, the better and vivid identification of the biliary tree following ICG application must be noted as a potential means of reducing bile duct injury. Thus the safety of laparoscopic cholecystectomy might be increased by employing ICG.

## Conclusion

Laparoscopic cholecystectomy with real-time indocyanine green fluorescence cholangiography enables a better visualization and identification of biliary tree and therefore should be considered as a means of increasing the safety of laparoscopic cholecystectomy.

## Abbreviations

ASA: American Society of Anesthesiologists
BMI: Body Mass Index
ICG: Indocyanine green
